# Stroke – prevalence, trends, and regional patterns in Germany. An analysis based on routine data from the statutory health insurance

**DOI:** 10.25646/13429

**Published:** 2025-09-24

**Authors:** Aline Anton, Alexander Rommel, Henriette Steppuhn, Roma Thamm, Dinara Yessimova, Helmut Schröder, Gabriela Brückner, Katrin Schüssel, Michael Porst

**Affiliations:** 1 Robert Koch Institute, Department of Epidemiology and Health Monitoring, Berlin, Germany; 2 AOK Federal Association, WIdO – AOK Research Institute, Berlin, Germany

**Keywords:** Stroke, Prevalence, Time trends, Morbidity, Age distribution, Health claims data, Secondary data analysis, Public Health

## Abstract

**Background:**

As part of the German Burden of Disease Study, population-based prevalences of important diseases are estimated. This allows regional patterns and temporal trends to be identified.

**Methods:**

The 10-year prevalence of stroke in the population was estimated cross-sectionally for the years 2017 to 2022 at the level of the Spatial Planning Regions using routine data of persons insured in the statutory health insurance AOK, adjusted for age, sex and morbidity (administrative 10-year prevalence).

**Results:**

In 2022, 1.4 % of the population in Germany was living with the consequences of a stroke that had occurred up to 10 years ago (women: 1.3 %, men: 1.6 %). Prevalence increases continuously with age – from 1.2 % (women) and 2.3 % (men) in the 60 – 64 age group to 8.3 % and 9.8 % in the 90 – 94 age group. Over time, age-standardised prevalence has remained largely stable since 2017. The age-standardised regional distribution shows a variation of up to 60 % between regions. Low values (below 1.1 %) are found mainly in southern German regions, while the highest values (above 1.5 %) without a clear pattern are found, for example, in the Emscher-Lippe, Saar, and southern Thuringia regions.

**Conclusions:**

Measured in terms of administrative 10-year prevalence, the significance of stroke for public health remains high, not least because of the high mortality rate, with high prevalence rates continuing to be achieved, especially in older age groups. In order to achieve a decline, more low-threshold, evidence-based prevention services are needed in addition to targeted interventions.

This article is part of a series of articles with standardised analysis for the German Burden of Disease Study of the Robert Koch Institute.

## 1. Introduction

In order to support health policy decisions, the evaluation of the burden of disease in the population is of growing importance. This involves using so-called summary measures of population health to compare diseases and injuries as causes of morbidity and mortality. Frequently used indicators are *years of life lost due to death* (YLL, mortality) and *years lived with disability* (YLD, morbidity), which are added together to form *disability-adjusted life years* (DALY) [1–4]. They represent the ‘loss’ of life years at the level of population health caused by health impairments and premature death. The methods were originally developed by the Global Burden of Disease Study (GBD) [4–6]. The GBD study was established in 1990 by the Harvard School of Public Health and the World Health Organization on behalf of the World Bank and is now conducted in the USA by the Institute for Health Metrics and Evaluation (IHME).

**Infobox Stroke**

A stroke is a sudden damage to areas of the brain, very often caused by a blockage in a blood vessel (cerebral infarction, ischemic stroke) or, in rarer cases, by a ruptured blood vessel and cerebral hemorrhage (hemorrhagic stroke). The health consequences of oxygen deprivation in the brain depend on the location and extent of the damage. Acute symptoms include paralysis, speech disorders, visual disturbances, balance disorders, and headaches. As the condition progresses, permanent cognitive impairments and limitations in activities of daily living (ADL) or self-care and mobility may occur [10]. Risk factors for stroke are diverse and include non-modifiable factors such as age, gender, and genetic predisposition, as well as modifiable factors such as high blood pressure, smoking, obesity, lack of exercise, diabetes mellitus, and lipid metabolism disorders [11, 12]. When considering gender differences, studies indicate that atrial fibrillation may be associated with a higher risk of more severe strokes in women, possibly due to a more unfavorable risk constellation, which underscores the need for gender-specific prevention measures for women [13].

Disease burden indicators make it possible to compare the impact of different diseases and to draw conclusions about regional differences and trends in population health over time. As part of the German Burden of Disease Study, this methodology is adapted and applied to diseases and injuries of high public health relevance. In the German pilot study (2018 – 2021), corresponding indicators were calculated for the reporting year 2017 [7, 8]. The selection of diseases is currently being expanded and updated based on data up to and including 2022.

Calculating the burden of disease requires reliable, comparable information on disease frequency by age, gender, and region over time. The prevalence rates used in this process also provide valuable insights for public health research, regardless of their use in determining the morbidity-related burden of disease (YLD). Routine data from statutory health insurance (SHI) providers offer a reliable basis for this for many diseases, including for small-scale analysis. Burden of disease studies thus provide important basic epidemiological information which, especially when carried out regularly, closes existing data gaps and supports the targeted planning and evaluation of prevention and care measures.

**Key messages**

► In 2022, 1.4 % of the population in Germany was living with the consequences of a stroke that had occurred up to 10 years earlier.
► The 10-year prevalence was 1.3 % in women and 1.6 % in men.
► The frequency increases significantly with age, with the highest prevalence among men at 9.8 % in the 90 to 94 age group.
► The regional distribution varies by up to 60 % between regions, with the lowest prevalence in southern and southwestern Germany and in some metropolitan areas.
► Over time, the administrative prevalence has remained largely unchanged since 2017.

Stroke is a major cause of disease burden in the population and is highly relevant to public health (Infobox). This article reports on the 10-year prevalence of stroke, which was determined as part of the Robert Koch Institute’s (RKI) disease burden study. It is based on the standard for reporting secondary data analyses in Germany [9].

In 2023, stroke (defined here as ICD-10: I60 – I64) ranked sixth among the most common causes of death in Germany and was responsible for approximately 3.4 % of all deaths [14]. According to the RKI’s national burden of disease study, strokes rank sixth among men and eighth among women (2017, DALY, Level 3), thus contributing significantly to the burden of disease in the population [7, 12].

Strokes are one of the most common reasons for hospitalisation. In 2023, 290,327 inpatient treatments due to stroke (ICD-10: I60 – I64 excluding I62; for definition see 2.2) were registered [15]. Early treatment in specialized stroke units in hospitals or mobile stroke units can significantly reduce the likelihood of sequelae in the first three months after a stroke [16, 17]. The long-term consequences of a stroke can lead to significant impairments in quality of life. The majority of those affected require intensive rehabilitation, which often lasts for months and requires interdisciplinary approaches [17]. Some of those affected leave rehabilitations with significant physical and cognitive deficits and are in some cases permanently dependent on care, which places a considerable psychosocial and financial burden on them and their relatives, as well as having economic consequences for the healthcare system [18, 19].

Over the past 25 years, a decline in stroke mortality has been observed [14, 20, 21], which is attributable to advances in acute care and thus to improvements in survival rates, especially for ischemic stroke [20–23]. However, this downward trend has slowed in the last decade. Reasons for the flattening trend in stroke mortality may lie in an increase in risk factors such as obesity and diabetes [20, 24–27]. Based on this development in combination with demographic changes in the population, an increase in stroke deaths is predicted in the coming years [20]. Between 2003 and 2014, the lifetime prevalence of stroke in adults increased by more than one percentage point from 1.9 % to 3.3 %, which is statistically significant. However, regionalized results are currently only available for five larger regions in Germany [28].

## 2. Methods

The present analysis is based on routine data of persons insured in the SHI system. These data are mainly generated by cost accounting between service providers (e.g. hospitals) and payers (health insurance funds) in the healthcare system and are only subsequently made available for research purposes (secondary data analysis). Routine SHI data are collected continuously and allow trend analyses as well as small-area analyses. The data contain the most important information for estimating the prevalence: (i) diagnoses according to the 10th revision of International Statistical Classification of Diseases and Related Health Problems (ICD-10-GM), (ii) services according to the official classification for the coding of surgeries, procedures and general medical measures (OPS) and (iii) drug prescriptions that can be categorised using the pharmaceutical central number (PZN) of the classification according to the Anatomical Therapeutic Chemical (ATC) system [29].

The underlying methodology for calculating prevalences based on routine SHI data consists of three steps: first, the definition of the prevalence concept in the insured population (see 2.1), second, the development of the case definition for identifying diseased persons (case selection criteria, see 2.2), and third, an age-, sex- and morbidity-adjusted extrapolation of the prevalences to the whole population using regression analysis. This allows statements to be made for all residents in the regions of Germany (see 2.3).

### 2.1 Insured population and prevalence concept of the 10-year prevalence of stroke

Pseudonymised routine data from around 27 million AOK insurance policyholders from the years 2017 to 2022 is analysed using a cross-sectional approach to identify people affected by a disease [30, 31]. Prevalence is defined as the proportion of persons affected by a disease during the analysis period out of the total number of people included in the study. In analyses using routine SHI data, it should be considered that the underlying population of insured persons is an open, dynamic cohort with inflows and outflows due to natural population movements (births, deaths) or changes in an individual’s insurance history (e.g. change of health insurance company). Therefore, all calculations are not based on individuals but on observed insurance periods from the respective reporting year in days [32]. In this way, insurance periods of new-borns or deceased persons, as well as those of persons who change insurance, can be considered on a pro rata basis. The period of insurance and the regional allocation of the insured is determined on a quarterly basis. Finally, the population of insured persons, and thus the denominator of the prevalence estimate, is obtained as the total number of observed quarterly insurance periods for the respective reference year [32, 33].

### 2.2 Case definition for stroke

A case definition for the inclusion of persons with prevalent stroke was developed in collaboration with renowned RKI internal and external experts (Table 1). The period analysed always refers to a 10-year prevalence in order to include survivors living with late neurological sequelae in the prevalence estimate. Those who died before the respective reporting year were not included in the prevalence calculation. Inclusion criteria are based on ICD-10-GM-coded diagnoses and from the inpatient care sector:

I62 (other non-traumatic intracranial hemorrhage) was not included because it differs from stroke both in its clinical picture (the neurological deficits typical of stroke) and in its lower degree of impairment and mostly uneventful recovery. Cases in all age groups were included in the prevalence calculation without restrictions, taking into account all persons in the insured population. The criteria were applied to all persons in all quarters of the reporting year, 39 quarters were looked back to give a total analysis period of 40 quarters for 10-year prevalences. Finally, to determine the number of persons affected by a disease and thus the numerator of the prevalence calculation, the observed person-time of the cases in each quarter of a calendar year was summed up.

### 2.3 Statistical methods

Since the group of policyholders of a health insurance fund is not a random sample of the general population and is therefore not representative of the population [31, 34–37], the specific prevalence estimates for each health insurance fund must be extrapolated to the whole population. Due to the regionally different distribution of the population in each health insurance fund, this extrapolation is done by region [38]. In this regression analysis, regionally available statistics on the frequency of inpatient diagnoses and on the demographic structure of the population on the level of the 400 German districts are used as auxiliary information. In this way, in addition to demographic differences, morbidity differences between health insurance funds and the German population can be corrected (morbidity-adjusted) and differentiated by small areas. The method was developed and its plausibility tested using type 2 diabetes as an example [38]. It has been adapted to estimate the prevalence for the whole population of Germany at the level of the 96 Spatial Planning Regions for stroke for each reference year.

When extrapolating prevalences, individual age groups are combined into larger age groups for model stability, so that a prevalence is not always available for each 5-year age group. To allow stratification at this level of detail, a special procedure is used to model missing age-specific prevalences. For this purpose, the sex-specific prevalence patterns of the AOK population along the 5-year age groups (raw data) are transferred to the (pooled) age groups of the extrapolation and then the resulting curves are smoothed using spline regression (using a B-spline) [39]. Point estimators that were extrapolated specifically for a 5-year age group using the morbidity adjustment procedure were excluded from the smoothing. In addition, the results are age-standardised using the European Standard Population 2013 [40] for the presentation of maps and time trends.

## 3. Results

**Figure 1:**
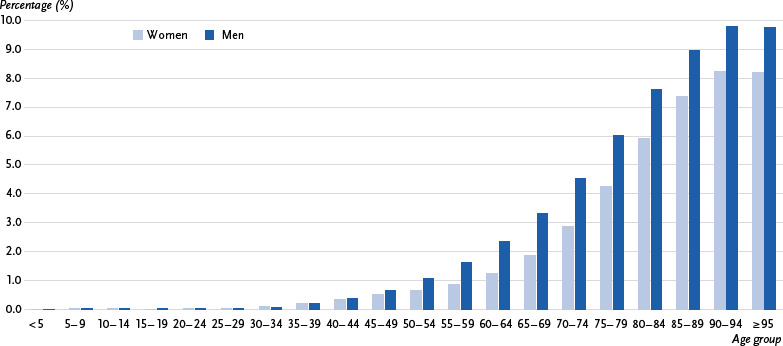
10-year prevalence of stroke by age and gender (percentage of population). Source: Burden of disease study for Germany (AOK routine data 2022, age-, sex- and morbidity-adjusted and extrapolated to the German population)

Based on the underlying case definition, in 2022 1.4 % of the population (around 1.2 million cases) in Germany had been affected by a stroke in the past ten years in 2022 (administrative 10-year prevalence). The prevalence was 1.3 % for women and 1.6 % for men. The prevalence of stroke increases significantly with age. While it is 0.4 % for people under 45, it rises from 2.6 % to 5.1 % for people between 65 and 79 years of age. From the age of 80 onwards, 6.6 % of adults are affected, with the prevalence reaching its highest level in the 90 to 94 age group at 8.3 % for women and 9.8 % for men (overall prevalence 8.7 %). There is a gender difference across almost the entire age range, with a prevalence difference of up to 1.8 percentage points lower in women (age group 75 – 79) (Figure 1 and Annex Table 1).

**Figure 2:**
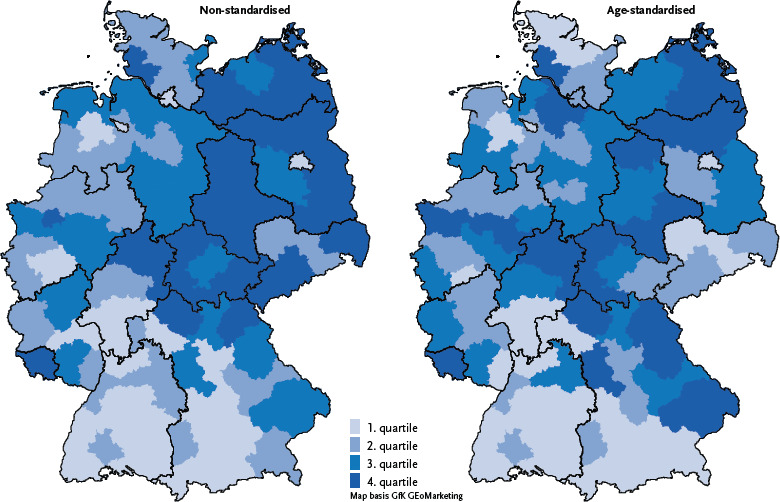
10-year prevalence of stroke on the level of the Spatial Planning Regions (percentage of the population, classification by quartiles). Source: Burden of Disease Study for Germany (AOK routine data 2022, age-, sex- and morbidity-adjusted and extrapolated to the German population)

The observed regional distribution of 10-year stroke prevalence shows small-scale differences with a northeast-south gradient (Figure 2, Annex Table 2). After age standardization, these differences flatten out significantly. The lowest age-standardised prevalences (1.0 – 1.1 %) for 2022 are found primarily in southern German (and partly southwestern German) regions such as Hochrhein-Bodensee and in some metropolitan regions (e.g., Munich, Rhine-Main, Stuttgart). The highest prevalence rates (1.5 – 1.6 %) occur without clear geographical accumulation, e.g., in the Emscher-Lippe, Saar, and South Thuringia regions. There, the values were approximately 0.6 percentage points higher than those of the regions with the lowest prevalence rates, which corresponds to a relative difference of approximately 60 %. In 2017, the variation or dispersion between regions was already at a similar level. Over time, there have been hardly any changes in regional differences.

**Figure 3:**
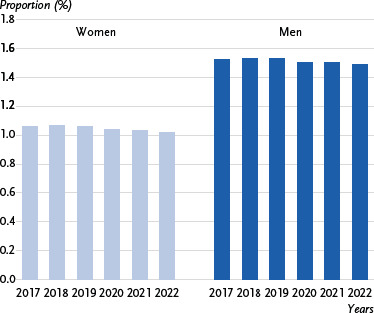
10-year prevalence of stroke over time (percentage of population, standardised by age). Source: Burden of Disease Study for Germany (AOK routine data 2017 – 2022, adjusted for age, sex and morbidity and extrapolated to the German population)

Over time, the overall 10-year prevalence of stroke (non-age-standardised) and age-standardised prevalence remained largely stable between 2017 and 2022. It fluctuated only slightly by 0.03 % (Figure 3 and Annex Table 3). There are no significant differences over time for either women or men.

## 4. Discussion

This analysis estimates the administrative 10-year prevalence of stroke in Germany based on routine SHI data and covers the years 2017 to 2022. The main results show that in 2022, approximately 1.4 % of the population in Germany had been affected by a stroke in the previous 10 years. This prevalence is higher in men (1.6 %) than in women (1.3 %) and increases significantly with age. Regionally, the administrative 10-year prevalence of stroke in 2022 is relatively heterogeneous. In most federal states, there are regions where it is significantly above the national average, while Baden-Württemberg and southern regions of Bavaria, among others, have below-average prevalence rates. There are relative differences of up to 60 % between the regional planning regions with the lowest and highest stroke prevalence. Over the period from 2017 to 2022, the administrative prevalence has remained largely stable.

Further estimates on stroke prevalence in Germany are available based on various definitions and studies. According to the results of the study ‘German Health Update’ (GEDA 2019/2020-EHIS), for example, 2.1 % of women and 2.3 % of men aged 18 and over stated that they had had a stroke in the last 12 months or were affected by chronic complaints as a result of a stroke [41]. Overall, the figures are therefore slightly higher than in the present study. Looking only at the older age groups, the figures in the present study for 2022 are 1.1 percentage points lower for women in the 65 – 79 age group and 1.2 percentage points higher for women in the 80 and older age group than in GEDA 2019/2020-EHIS (2.8 % vs. 3.9 % and 6.7 % vs. 5.5 %). For men, on the other hand, the values are 1.8 percentage points lower in the 65 – 79 age group, while they are 2.3 percentage points higher in the 80 and older age group than in the GEDA evaluation (4.4 % vs. 6.2 % and 8.2 % vs. 5.9 %) [41]. Reasons for the observed differences in the older age groups may lie in distortions due to selection and accessibility effects: While those affected in the 65 – 79 age group are easier to reach for participation in surveys, routine data in the 80 and older age group capture prevalence more systematically, as they also include institutionalized and care-dependent individuals in their entirety. Compared to the results of the Health Atlas [42], which are also based on routine data, the results on age- and gender-related prevalence are only minimally lower because the ICD code I62 (other non-traumatic intracranial hemorrhage) was not taken into account for the present evaluations. Furthermore, the prevalence data are not directly comparable because the Health Atlas uses a different reference population (persons aged 20 and over). Overall, the results of the routine data analyses presented here are consistent in magnitude with the prevalence rates determined in earlier studies, although methodological differences, particularly in case definition and data basis (self-reported data vs. routine data), could explain slight deviations.

Compared to international prevalence estimates, the values presented here show some deviations. For example, the Global Burden of Disease (GBD) study reports a total prevalence of 2.4 % for Germany in 2021 (women: 2.4 %, men: 2.5 %) [12]. However, the GBD methodology uses a case definition with a broader list of ICD codes. In addition, the GBD methodology is based on global modeling and also takes into account data from countries with widely differing health care systems and diagnostic standards, which can lead to deviations [6]. The present study uses national SHI routine data and considers specific regional and demographic adjustments in order to validly estimate the administrative 10-year prevalence for the total population in Germany, which increases its significance for Germany.

The regional variation in stroke prevalence of up to 0.6 percentage points across Germany can potentially be attributed to a combination of non-demographic factors such as health behaviour (e.g., smoking, obesity, and physical inactivity) and differences in prevention, care, and socioeconomic conditions. Studies by the RKI show that socioeconomic disadvantage is associated with a higher prevalence of stroke. In particular, people in the low education group are about three times more likely to suffer a stroke than people in the medium and high education groups, with the differences being more pronounced in women than in men [20, 41]. In addition, there are correlations between spatial socioeconomic disadvantage, such as the accessibility of stroke units, and stroke mortality [43]. Further analysis is needed to identify the factors that explain the observed regional differences in stroke prevalence. Once the data on disease burden is available, a comparison between the prevalences shown here and the DALY disease burden indicator based on them, in combination (as a bivariate map) with the socioeconomic index (German Index of Socioeconomic Deprivation, GISD [44]), can also contribute to a more comprehensive regional assessment.

Following the observed increase in stroke prevalence in nationwide health survey data up to 2014 [28], the present results based on routine SHI data for the years 2017 – 2022 indicate that prevalence rates have recently stabilized over time. Reasons for this development in stroke prevalence may be related to the development of risk factors. An improvement in blood pressure values and total cholesterol and triglyceride levels measured in blood serum, which was observed on the basis of nationwide examination surveys of adults in Germany (from 1990 to 2011) [45–47], can be viewed positively. However, surveys since 2003 have observed an increase in the prevalence of diabetes [27] and obesity [26]. Two studies show that many adults, including those with non-communicable diseases such as stroke, do not meet the WHO’s recommended minimum requirements for physical activity. One of the studies highlights that despite a certain increase in physical activity, many people, especially those with pre-existing health conditions, remain insufficiently active [48]. According to GEDA 2019/2020-EHIS, 46.6 % of adults in Germany do not meet the WHO recommendation of at least 150 minutes of moderate to vigorous physical activity per week [49]. With regard to smoking behaviour in the population, after a slow but steady decline in smoking prevalence between 2003 and 2023, there were signs of stagnation from 2019 onwards, particularly in the prevalence of daily smoking [26]. According to a nationwide survey by the RKI, 28.9 % of adults in Germany smoked tobacco products daily or occasionally in 2019 [50]. The smoking rate in Germany is also high compared to other European countries [51]. In addition, the decades-long decline in stroke mortality has flattened significantly over the past decade [24]. This goes hand in hand with a recent stagnation in life expectancy in Germany [43, 52, 53], which, like the incidence of stroke, is linked to socioeconomic inequality. Premature deaths from cardiovascular diseases such as stroke and coronary heart disease contribute significantly to a reduction in life expectancy, especially in the most socioeconomically disadvantaged groups [54, 55].

The present analyses are based on routine data from statutory health insurance funds. They provide a valid basis for estimating the prevalence of serious diseases such as stroke, particularly due to the high number of cases and the complete recording of diagnoses in the inpatient sector. Routine data also have the advantage that some typical sources of error in primary data collection, such as surveys, are excluded. These include distortions due to recall bias, non-response, or lower participation rates among groups of people who are difficult to reach [56]. One limitation to consider is that routine SHI data primarily contains information that is relevant for cost accounting (see 2. Methods). Non-use of the healthcare system, lack of documentation of diagnoses, and coding errors can lead to misclassifications and distortions in the data [56, 57]. Non-use is of little relevance for many diseases if they are so serious, such as strokes, that they usually lead to medical contact or hospitalization. Further limitations of the results are based on the statistical methods used to extrapolate [38] to the total population and to model age progression in 5-year age groups. The extrapolation method uses the diagnosis frequencies of all inpatient cases in hospitals in Germany to adjust for health insurance-specific morbidity differences in the population and was developed and validated using the example of type 2 diabetes [38]. By analogy, it is therefore also assumed for strokes that the estimated prevalences no longer reflect health insurance-specific morbidity, but rather that of the population. To model age progression, it was assumed that the age progression of stroke prevalences among AOK-insured persons could be transferred to the combined age groups from the extrapolation results. In order to verify the plausibility of the results obtained from the extrapolation, the modelling of age trends, and the smoothing procedure, they were compared with published values for stroke prevalence in Germany, whereby the deviations were minor or attributable to methodological differences in the case definitions [58, 59].

Cardiovascular diseases such as stroke remain one of the key challenges for public health and the healthcare system. The age- and gender-related differences in stroke prevalence underscore the need for life-stage-specific and gender-related prevention approaches. Research and practice should continue to focus more on the prevention of modifiable risk factors such as high blood pressure, smoking, and physical inactivity [60, 61]. Medical guidelines can also contribute to evidence-based prevention, such as the S3 guideline on cardiovascular prevention risk counseling by general practitioners, which is funded by the Innovation Fund [62]. In addition, stroke registries make it possible to collect data on epidemiology, risk factors, and stroke care and to report on incidence and long-term outcomes; one example is the population-based Erlangen Stroke Registry [23]. At the same time, it is important to continue analysing the causes of regional differences in prevalence in order to identify gaps in prevention and care and to plan targeted measures. The regular collection of reliable data on stroke risk factors is highly relevant for the development of appropriate interventions. Given the high proportion of stroke survivors who require long-term care, the further development of sustainable rehabilitation and support services is also crucial.

## Figures and Tables

**Table 1:** Selection criteria for defining the prevalence of stroke using routine AOK data

Care sector	Inpatient care^1^
Inclusion criterion	
**Criterion**	At least one primary diagnosis in the 10-year analysis period (40 quarters)
**Codes**	ICD-10-GM: I60, I61, I63, I64 I60: Subarachnoid haemorrhage I61: Intracerebral haemorrhage I63: Cerebral infarction I64: Stroke, not specified as hemorrhage or infarction

^1^ Inpatient cases (Section 301(1) SGB V): Main diagnosis of completed inpatient and day-care cases (discharge diagnoses)

ICD-10-GM = International Statistical Classification of Diseases and Related Health Problems, 10th Revision, German Modification, SGB = Social Security Code

**Annex Table 1:** 10-year prevalence of stroke by age and gender (percentage of population). Source: Burden of Disease Study for Germany (AOK routine data 2022, adjusted for age, gender, and morbidity and extrapolated to the population of Germany)

Age group (years)	Women	Men	Total
	%	%	%
00 – 04	0.01	0.01	0.01
05 – 09	0.03	0.03	0.03
10 – 14	0.03	0.03	0.03
15 – 19	0.01	0.02	0.02
20 – 24	0.02	0.02	0.02
25 – 29	0.04	0.03	0.03
30 – 34	0.10	0.08	0.09
35 – 39	0.21	0.19	0.20
40 – 44	0.35	0.38	0.37
45 – 49	0.50	0.66	0.58
50 – 54	0.66	1.06	0.86
55 – 59	0.87	1.61	1.24
60 – 64	1.24	2.35	1.79
65 – 69	1.87	3.32	2.56
70 – 74	2.86	4.54	3.64
75 – 79	4.27	6.02	5.05
80 – 84	5.90	7.61	6.62
85 – 89	7.36	8.97	7.97
90 – 94	8.25	9.81	8.73
≥ 95	8.19	9.77	8.54
Total	1.32	1.56	1.44

**Annex Table 2:** 10-year prevalence of stroke on the level of the Spatial Planning Regions (percentage of the population), classification by quartiles. Source: Burden of Disease Study for Germany (AOK routine data 2022, age-, sex- and morbidity-adjusted and extrapolated to the German population)

		Not standardised	Age-standardised
4. quartile	beyond	1,66	1,34
3. quartile	to	1,66	1,34
2. quartile	to	1,49	1,26
1. quartile	to	1,34	1,18

**Annex Table 3:** 10-year prevalence of stroke over time (raw and age-standardised percentage of population). Source: Burden of Disease Study for Germany (AOK routine data 2017 – 2022, adjusted for age, gender, and morbidity and extrapolated to the population of Germany)

Year	Women (not standardised)	Men (not standardised)	Total (not standardised)	Women (age-standardised)	Men (age-standardised)	Total (age-standardised)
	%	%	%	%	%	%
2017	1.34	1.52	1.43	1.06	1.52	1.27
2018	1.35	1.55	1.45	1.07	1.53	1.28
2019	1.36	1.57	1.46	1.06	1.53	1.27
2020	1.34	1.56	1.45	1.04	1.50	1.25
2021	1.34	1.56	1.45	1.03	1.50	1.24
2022	1.32	1.56	1.44	1.02	1.49	1.24
